# A case of breast cancer involving a ventriculoperitoneal shunt

**DOI:** 10.1186/s40792-016-0136-7

**Published:** 2016-02-06

**Authors:** Mirei Kamei, Nobuyuki Kikuchi, Homare Ichimura, Masao Chujo, Yoshiaki Takahashi, Kenji Sugio

**Affiliations:** Department of Surgery, Shin-beppu Hospital, 3898 Tsurumi, Beppu, Oita 874-5593 Japan; Department of Neurosurgery, Shin-beppu Hospital, Beppu, Oita 874-5593 Japan; Kitsuki Central Hospital, 120 Kitsuki, Kitsuki, Oita Japan; Department of Thoracic and Breast Surgery, Faculty of Medicine, Oita University, 1-1 Idaigaoka, Hasama, Yufu, Oita 879-5503 Japan

**Keywords:** Breast cancer, Ventriculoperitoneal shunt, Shunt malfunction

## Abstract

An 84-year-old woman was examined for an enlargement of an induration in the left breast. A ventriculoperitoneal shunt had been placed for postoperative normal pressure hydrocephalus of a cerebral hemorrhage, and it had penetrated the mass according to the computed tomography findings. Breast cancer was diagnosed after a close examination; however, close observation was selected because her family rejected treatment. She developed somnolence 7 months after the initial examination, and ventricular dilatation and expansion of the low-density region around the ventricle were noted on computed tomography, suggesting that the enlarged tumor had excluded the shunt and caused obstruction. The growth of breast carcinoma involving a shunt tube can be the cause of obstruction of a ventriculoperitoneal shunt. Our findings suggest that a breast lesion should be evaluated at both pre- and postoperation.

## Background

Ventriculoperitoneal (VP) shunt operation is the common neurosurgical procedure for hydrocephalus. Although a VP shunt is frequently associated with complications, the complications related to the breast are rare according to the previous literature. These include cerebrospinal fluid (CSF) pseudocysts, CSF galactorrhea, and the migration of the shunt tube inside the silicone breast implant. We herein present a case of breast cancer involving a VP shunt with a review of the pertinent literature.

## Case presentation

**Fig. 1:**
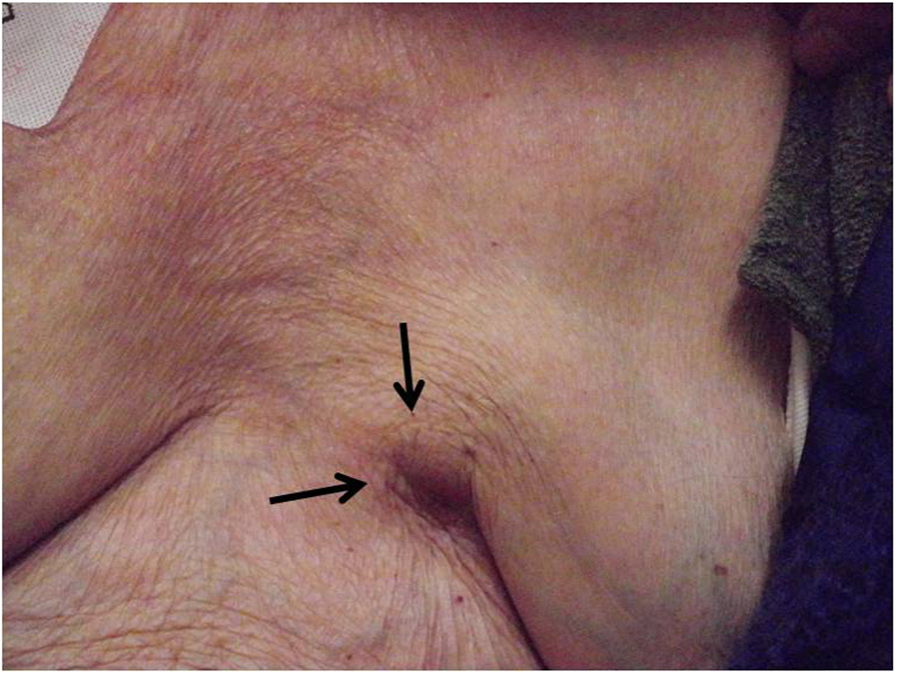
A mass accompanied by redness was present in the medial region of the left breast

**Fig. 2:**
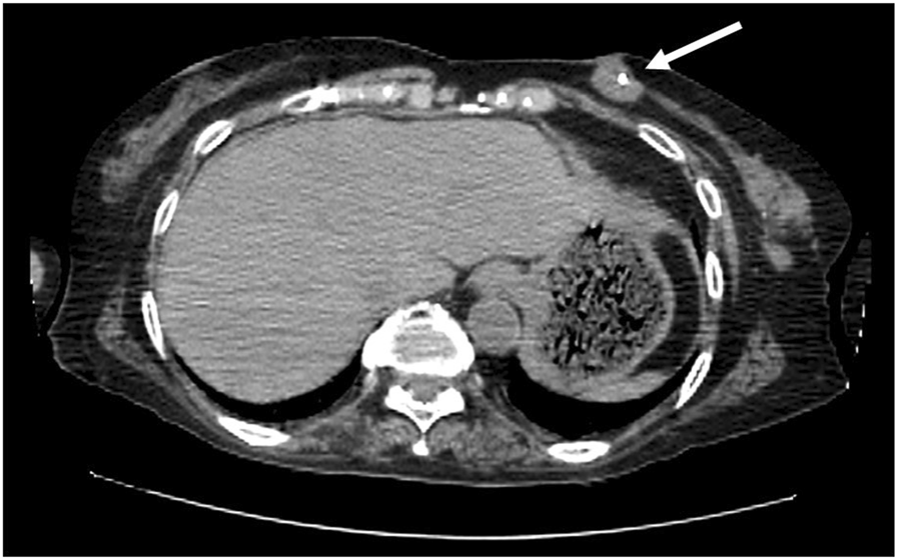
A CT scan showed that the tumor involved the VP shunt

**Fig. 3:**
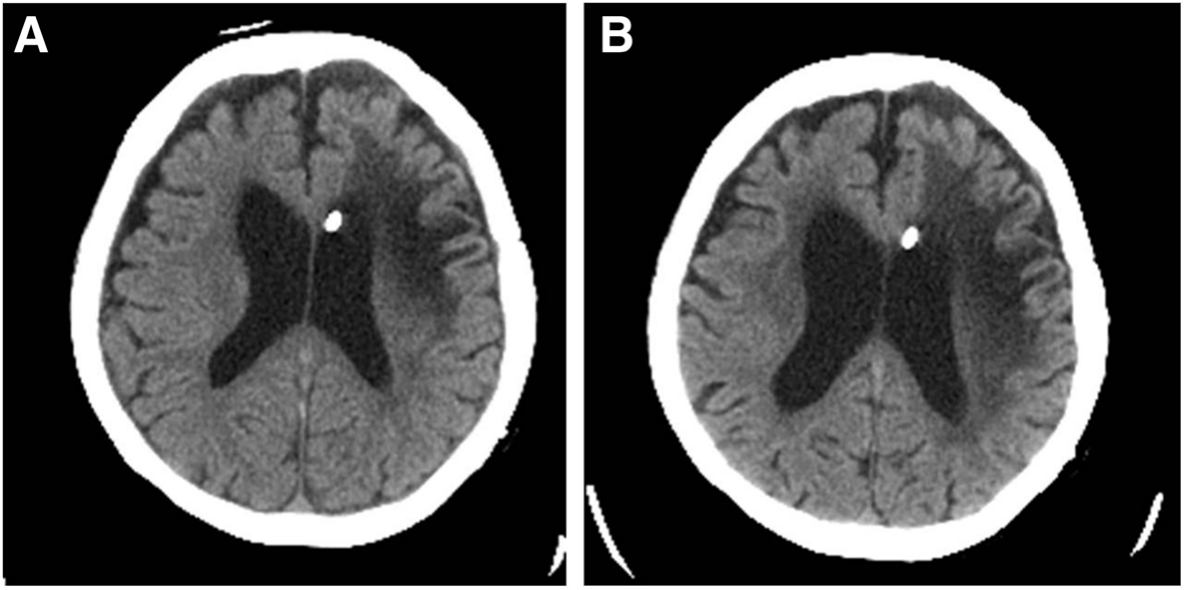
Brain CT showed that the ventricle was more dilated and the low-density region around the ventricle had expanded within 7 months (**b**) after the initial visit to our hospital (**a**)

The patient was an 84-year-old woman in whom a VP shunt had been placed for normal pressure hydrocephalus after a cerebral hemorrhage at 69 years of age, and she was in a state of dysbasia with an Eastern Cooperative Oncology Group (ECOG) performance status (PS) of 3. While staying in a facility, a mass in the left breast had been noted on a visiting examination by the primary care hospital staff. Since the mass was enlarged, she was examined by a physician. A 3-cm hard mass retracting the dark red skin was palpated on the medial side of the left breast (Fig. 1). Differentiation of a subcutaneous malignant tumor was also considered; however, left breast cancer was suspected according to the ultrasonography findings, and fine needle aspiration cytology (FNAC) was performed. The cytological findings showed increases in the nucleus/cytoplasm (N/C) ratio and chromatin content, an aggregation of atypical cells, and intracytoplasmic lumen. According to these findings, the mass was diagnosed as infiltrating breast cancer. The carcinoembryonic antigen (CEA) level was 8.0 ng/ml, cancer antigen15-3 (CA15-3) was 42.2 U/ml, and breast carcinoma-associated antigen225 (BCA225) was 210 U/ml, all of which were elevated. On whole-body computed tomography (CT), the mass was observed to involve the VP shunt (Fig. 2) and infiltrated the mammary gland over the skin; however, axillary lymph node metastasis and distant metastasis were not observed. Because damage to the shunt due to a needle puncture was a concern, the patient was referred to the neurosurgery department of our hospital; however, there was no problem with the shunt. En bloc resection of the VP shunt and breast was recommended to prevent problems with the shunt, but close observation was selected because her family rejected any treatment. At 7 months after the initial examination, the tumor marker levels were higher: CEA, 9.5 ng/ml; CA15-3, 56 U/ml; and BCA225, 200 U/ml. Skin redness and lethargy developed, and tumor enlargement was observed. A CT scan of the head showed dilated ventricles and the enlargement of periventricular lucency, suggesting that the tumor enlargement caused the obstruction (Fig. 3a, b). Three months later, the patient became bedridden and suffered from aspiration pneumonia due to dysphagia.

### Discussion

Hydrocephalus can be developed in all generations. Many hydrocephalus patients receive a CSF shunt, which is applied to 16,000 patients annually in Japan, and a VP shunt is most frequently placed. The average lifespan of Japanese women has exceeded 80 years, and the elderly population is increasing. Therefore, the number of shunt operations performed annually is likely to increase due to an increase in elderly patients. On the other hand, the incidence of breast cancer has continuously increased in Japan. The number of breast cancer patients with CSF shunts may be increasing. Complications frequently associated with a VP shunt, including shunt obstruction, infection, overdrainage of CSF, and perforation of the gastrointestinal tract, gallbladder, vagina, and abdominal wall at the umbilicus, have also been reported [1]. Regarding complications associated with the mammary gland, CSF pseudocysts due to the leakage of CSF from the catheter shunt obstruction, CSF galactorrhea, and shunt migration of the VP shunting catheter under or within silicone breast implants have been previously reported; however, they are considered to be very rare [2–5].

In the present patient, breast cancer involving a VP shunt was observed, which is very rare and only three cases have been previously reported in the English literature [6–8] (Table 1). All patients had advanced age (67 to 88 years of age). As a person ages, atrophy of the breast become progressive and fat tissue replaces the breast tissue. Therefore, a shunt can easily insert into the breast tissue that has been replaced with fat tissue in cases of shunt operation. We speculate that this is the cause of breast-related shunt complications. Since a silicone catheter is very soft, it is unlikely that the shunt catheter pierced the breast cancer. The cancer may have developed and grew around and involved the shunt catheter. In the published literature, only one case of breast cancer demonstrated neurologic symptoms due to shunt trouble [6]. This previous case was detected at screening without symptoms; in another case and our case, a skin lesion was noted without other neurologic symptoms [7, 8]. Therefore, it is important to perform breast cancer screening before and after the shunt operation. FNAC was performed for the diagnosis of breast cancer in three cases including our case. In our case, FNAC was performed before CT screening, while in the other two cases, it was performed after CT screening or magnetic resonance imaging (MRI) scan. Therefore, the positional relationship between the tumor and shunt tube should be confirmed before aspiration in cases in which the shunt tube and tumor are on the same side of the chest.

**Table 1:** Cases in the literature of breast cancer involving a VP shunt

Author	Age (years)	Past history	Region of the tumor	Tumor size	Neurologic symptoms (other symptoms)	Examination of the tumor	Treatment
Roka [6]	70	NPH	Rt. upper inner quadrant	8.0 cm	Headache, drowsiness, altered sensorium	FNAC	Radical mastectomy/rerouting of the VP shunt
Lee [7]	88	Head trauma	Rt. upper inner quadrant	1.7 cm	No symptoms (hard skin lesion)	Biopsy of skin	No operation/hormone therapy
Jain [8]	67	Ependymoma	Rt. upper inner quadrant	1.3 cm	No symptoms (breast screening)	FNAC	Wide local excision, SLNB/rerouting of the VP shunt
Present case	84	Cerebral hemorrhage	Lt. under inner quadrant	3.0 cm	No symptoms (hard skin lesion)	FNAC	No treatment

*NPH* normal pressure hydrocephalus, *FNAC* fine needle aspiration cytology

Roka et al. reported consciousness disturbance due to hydrocephalus symptoms induced by catheter obstruction by a breast cancer mass [6]. Our case was not treated, and stenosis by catheter compression occurred 7 months after the initial examination. The ventricle was dilated and periventricular lucency expanded, suggesting the aggravation of hydrocephalus. However, the PS and consciousness of our patient was poor from the first visit, thus it was difficult to determine worsening of her condition, except that she was somnolent and bedridden. If the breast cancer was discovered earlier, there might be a possibility that the shunt tube had not been involved with breast cancer. Taken together, partial resection of the breast under local anesthesia may be a treatment of choice leading to convince her family. Therefore, this outcome may have been changed.

Regarding breast cancer operation, resection of the breast cancer, including shunt placement and rerouting of the shunt, is necessary to prevent dissemination through the catheter by a breast surgeon and neurosurgeon in close cooperation with one other. There was no effective means to treat this case, while her family did not want us to perform any invasive treatments or diagnostic procedures. However, endocrine therapy would have been one treatment option for this patient. Various diagnostic procedures, including US-guided biopsy and incision biopsy should be performed in such cases to obtain tissue samples and thereby identify the intrinsic subtype. For cases with skin involvement, intervention can also be performed without damaging the shunt tube in order to obtain subcutaneous tissue that may have become infiltrated with cancer cells. Even if such tissue sampling proves to be impossible, treating such patients with endocrine therapy is still one therapeutic alternative.

In any case, regular breast cancer screening after shunt operation is necessary.

## Conclusions

We herein encountered a very rare case of breast cancer involving a VP shunt. In Japan, the incidence of breast cancer and the number of elderly individuals have increased. An evaluation for breast cancer before shunt placement, in addition to an examination of the body surface, and follow-up breast cancer screening after shunt placement is necessary.

## Consent

Written informed consent was obtained from the patient for publication of this case report and any accompanying images. A copy of the written consent is available for review by the Editor-in-Chief of this journal.

## Abbreviations

BCA225: breast carcinoma-associated antigen225
CA15-3: cancer antigen15-3
CEA: carcinoembryonic antigen
CSF: cerebrospinal fluid
CT: computed tomography
ECOG: Eastern Cooperative Oncology Group
FNAC: fine needle aspiration cytology
MRI: magnetic resonance imaging
N/C: nucleus/cytoplasm
PS: performance status
VP shunt: ventriculoperitoneal shunt
